# Development and validation of a prognostic scoring system for patients with colorectal cancer hepato-pulmonary metastasis: a retrospective study

**DOI:** 10.1186/s12885-022-09738-3

**Published:** 2022-06-11

**Authors:** Shenghe Deng, Zhenxing Jiang, Yinghao Cao, Junnan Gu, Fuwei Mao, Yifan Xue, Le Qin, Ke Liu, Jiliang Wang, Ke Wu, Kailin Cai

**Affiliations:** Department of Gastrointestinal Surgery, Union Hospital, Tongji Medical College, Huazhong University of Science and Technology, Wuhan, 430022 Hubei China

**Keywords:** Colorectal cancer, Hepato-pulmonary metastasis, Nomogram, Overall survival, Cancer specific survival, Prognosis

## Abstract

**Background:**

Hepato-pulmonary metastasis of colorectal cancer (CRC) is a rare disease with poor prognosis. This study aims to establish a highly efficient nomogram model to predict overall survival (OS) and cancer-specific survival (CSS) in patients with colorectal cancer hepato-pulmonary metastasis (CRCHPM).

**Methods:**

We retrospectively analyzed the data of patients with CRCHPM from SEER database and Wuhan Union Hospital Cancer Center (WUHCC). A total of 1250 CRCHPM patients were randomly assigned to the training, internal validation, and external validation cohorts from 2010 to 2016.Univariate and multivariate cox analysis were performed to identify independent clinicopathological predictors of OS and CSS, and a nomogram was constructed to predict OS and CSS in CRCHPM patients.

**Results:**

A nomogram of OS was constructed based on seven independent predictors of age, degree of differentiation, T stage, chemotherapy, number of lsampled lymph nodes, number of positive lymph nodes, and tumor size. Nomogram showed favorable sensitivity in predicting OS at 1, 3 and 5 years, with area under the receiver operating characteristic curve (AUROC) values of 0.802, 0.759 and 0.752 in the training cohort;0.814, 0.769 and 0.716 in the internal validation cohort;0.778, 0.756 and 0.753 in the external validation cohort, respectively. A nomogram of CSS was constructed based on three independent predictors of T stage, chemotherapy, and tumor size. The AUROC values of 1, 3 and 5 years were 0.709,0.588,0.686 in the training cohort; 0.751, 0.648,0.666 in the internal validation cohort;0.781,0.588,0.645 in the external validation cohort, respectively. Calibration curves, Concordance index (C-index), and decision curve analysis (DCA) results revealed that using our model to predict OS and CSS is more efficient than other single clinicopathological characteristics.

**Conclusion:**

A nomogram of OS and CSS based on clinicopathological characteristics can be conveniently used to predict the prognosis of CRCHPM patients.

## Background

Colorectal cancer (CRC) is the third most common cancer worldwide, with an average 5-year OS rate of 60%, and during the metastatic phase, OS is significantly reduced [1]. Advanced colorectal cancer is still a fatal disease. Despite improvements in survival rates at diagnosis of metastatic disease over the past 20 years, significant heterogeneity in survival outcomes persists [2]. With the change of living habits and diet, the prevalence of CRC is increasing and the incidence is gradually younger [3]. In February 2022, the National Cancer Center released the latest issue of cancer statistics in China. In general, the incidence of CRC is 10.67% in men and 9.26% in women, and the overall incidence of CRC is 10.04% [4]. Feng et al. [5] analyzed the current cancer situation in China according to the data provided by Cancer Today Institute in 2018. The incidence of CRC is 12.8% in men, that 11.3% in women, and the overall incidence of CRC is 12.2%. CRC is one of the cancers with high morbidity and mortality in China. In addition, CRC is also a cancer with a high rate of metastasis, with distant metastasis further reducing patient survival. Population-based studies have shown that approximately 25–30% of patients diagnosed with CRC developed liver metastases during the course of the disease [6, 7]. The lung is the second most common site of metastasis for CRC second only to the liver. Compared with colon cancer, rectal cancer has a higher incidence of lung metastases. In China, the percentage of rectal cancer cases is about 50%, much higher than in Western countries nearly 30%, the burden of cancer has increased significantly [8].

Although the OS of CRC patients with distant metastasis is poor, the prognosis of patients with metastatic colorectal cancer has improved over the past few years due to improved surgical techniques, enhanced perioperative care, and huge developments in treatment modalities. In particular, patients with unresectable liver or lung metastasis at the initial stage can achieve tumor reduction through various chemotherapy methods, thus achieving tumor resection and improving survival rate [8–11]. In the 1990s, 2-year OS for stage IV colorectal cancer was only 21%, but in the 2010s, 5-year OS increased to 35–40% [12, 13]. At present, the prognosis of patients with metastatic CRC has been improved, however, it is mainly for CRC patients with single organ metastasis, and the prognosis of CRC patients with multiple organ metastasis is still poor [14, 15].

Patients with hepato-pulmonary metastases from CRC are rare and have a poor prognosis, which can lead to a significant decrease in survival rates with various treatment modalities and thus place a significant burden on the healthcare system. Our inability to accurately predict prognosis has been identified as a significant barrier to effective physician-patient communication [16]. Therefore, accurately predicting the prognosis of patients with CRCHPM is crucial for treatment selection and effective patient-provider communication. However, it is difficult to predict the OS for mixed advanced cancer patients with limited life expectancy and poor physical states [17].

The prognosis of CRC is related to lymph nodes, TNM stage, radiotherapy, and primary location of the tumor, but individual indicators are not sufficiently predictive of prognosis. Engstrand J et al. [18] used cox regression to predict survival rates and showed that survival rates after liver metastases in CRC varied by location. Tang et al. [19] identified 11 prognostic factors affecting liver metastasis from CRC by creating a nomogram and suggested that gender is not only a risk factor but also a prognostic correlate of liver metastasis from CRC. In our previous studies, we also found that a nomogram constructed based on clinicopathological characteristics could be easily used to predict OS and CSS in patients with liver metastasis from CRC [20]. In addition, related studies have also predicted the prognosis of patients with CRC lung metastasis based on clinicopathological characteristics. Huang et al. [21] analyzed clinicopathological characteristics by multivariate logistic regression analysis and found that 9 clinicopathological characteristics were independent risk factors for lung metastasis of CRC, and nomogram construction based on these indicators can effectively predict prognosis. Wang et al. [22] constructed a nomogram model combining machine learning-pathology, radiological features, immune score and clinical factors to predict the prognosis of CRC patients after lung metastasis, and found that this nomogram could be used to predict OS and DFS after lung metastasis of CRC patients. However, these studies only predicted the prognosis of patients with single organ metastasis of CRC, and did not study the prognosis of patients with multiple organ metastasis of CRC. To our knowledge, there are few studies on the prognosis of CRC with multiple organ metastasis, and there is a lack of their own clinical data to verify [23, 24]. Due to the poor prognosis of CRCHPM, the study on the prognosis of CRCHPM deserves further exploration.

In our study, patients with CRCHPM were divided into different cohorts based on the public database and the data of our medical center. Then accurate and effective OS and CSS prediction nomograms of patients with CRCHPM were established according to clinicopathological characteristics, and the predictive value of the prediction nomograms was further evaluated.

## Methods

### Study design and data collection

**Fig. 1:**
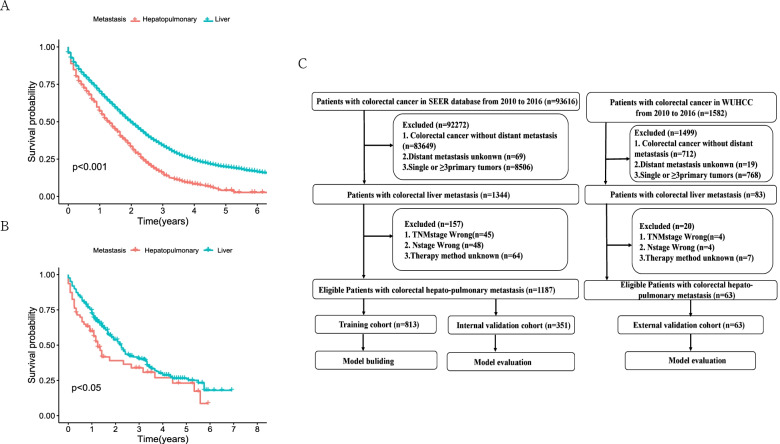
Patients with CRCHPM OS and study design. Overall survival difference between CRCHPM and CRCLM patients in SEER database (**A**) and WUHCC cohort (**B**); and strategies for selecting patients for inclusion in the study (**C**)

In this study, a total of 1250 patients with CRCHPM were enrolled, including 1187 from the SEER dataset and 63 from the WUHCC cohort. The selection criteria included: CRC patients diagnosed with hepato-pulmonary metastasis at all ages from 2010 to 2016.A detailed flow diagram of the patient selection process is shown in Fig. 1.

Age, sex, tumor location, degree of differentiation, histological type, tumor size, TNM, T stage, N stage, M stage, radiotherapy, chemotherapy, number of sample lymph nodes, number of positive lymph nodes, primary surgery, and metastatic surgery were analyzed in all three cohorts. Patients in the SEER database were randomly divided into training cohort and internal validation cohort, and eligible patients from the WUHCC cohort were used as external validation cohort. The model was established according to the training cohort, and then the model was verified by two validation cohorts. The study protocol was reviewed and approved by the Ethics Committee and institutional Review Committee of Wuhan Union Hospital (No.2018-S377). All patients provided written informed consent and all procedures performed in studies involving human participants were accordance with the Helsinki Declaration.

### Nomogram establishment and validation

Univariate and multivariate cox regression analyses were used to calculate the impact of each clinicopathological characteristics on OS and CSS in the training, internal and external validation cohorts. The impact of each variable on CSS and OS was measured as a hazard ratio (HR) and used to identify independent prognostic risk factors. A nomogram was established based on the independent prognostic clinicopathological characteristics obtained by training cohort multivariate cox regression. The total points of each patient in the training, internal validation and external validation cohorts was calculated according to the established nomogram, and cox regression analysis was performed on the three cohorts using the total points as a parameter.

### AUROC, calibration curve, C-index and DCA analysis

The results of nomogram calibration are evaluated by Hosmer-Lemeshow test and displayed in the form of calibration curves. The ROC curves of 1-, 3- and 5 years are used to show the accuracy of nomogram’s time prediction ability, and AUROC was used to evaluate nomogram’s ability to predict OS and CSS in CRCHPM. In addition, c-index was defined as the ratio of all patient pairs predicted to conform to the results, DCA has recently been proposed as a new way to visualize the potential clinical value of risk prediction models. Therefore, the above methods were adopted in this study to verify the clinical efficacy of nomogram.

### Statistical analysis

Categorical variables were expressed as number and percentage and Chi-square test or Fisher’s exact test was used for comparison. Univariate and multivariate cox regression analysis was used to calculate the influence of variables on OS and CSS. The impact of each variable was evaluated by HR. In the multivariate cox regression model, the method of stepwise selection is adopted to filter the variables (*p* < 0.05). Statistical Package for the Social Sciences (SPSS) 22.0 (IBM Corp., Armonk, NY, USA) and R 4.0.2 (R Foundation for Statistical Computing, Vienna, Austria) were used for Statistical analysis. The R packages used were “rms”, “survival”, “survminer”, “regplot”, “ggDCA”, “timeROC” and “survcomp”. All statistical tests were two-sided, and the statistical significance was set to 0.05.

## Results

### Prognosis and clinicopathological characteristics of patients with CRCHPM

First, we analyzed the OS of CRCHPM and colorectal cancer liver metastasis (CRCLM) in SEER database and WUHCC cohort from 2010 to 2016. 1187 and 9332 patients with CRCHPM and CRCLM occurred in SEER database, respectively.63 and 340 patients in WUHCC cohort respectively. And we found that the OS of patients with CRCHPM was significantly worse than that of patients with CRCLM in both cohorts (Fig. 1A, B). Subsequently, patients with CRCHPM were analyzed. In the SEER database, 70% (*n* = 831) of patients were randomly assigned to the training cohort, while the remaining patients (*n* = 351) were included in the internal validation cohort. Patients (*n* = 63) from the WUHCC cohort were included in the external validation cohort. The detailed flow diagram of the standard procedures for patient inclusion and exclusion is shown in Fig. 1C.

The mortality was 81.6 and 66.76% for all CRCHPM patients in the SEER and WUHCC cohorts, respectively. Most patients were older than 60 years (692,58.2%;41;65.1%) and male (632,53.2%;33,52.4%) in the SEER cohorts and WUHCC cohorts, respectively. In the SEER cohorts, compared with alive patients, dead patients were more likely to have larger tumor diameter, higer T stage, higher rates of postive lymph node and tumors localized more commonly in the colon. In the WUHCC cohorts, dead patients were more likely to have higer N satge, higher rates of sampled lymph node metastasis and tumors localized also more commonly in the colon. In both the SEER and WUHCC cohorts, most patients received neoadjuvant chemotherapy (70.6 and 65.1%, respectively), while fewer patients received radiotherapy (6.0 and 9.5%, respectively, Table 1).

**Table 1:** Clinicopathological characteristics of patients in the training, internal validation and external validation cohorts (N(%))

Characteristics	SEER			SEER			HUST-JU		
	Training cohort		*P*	Internal validation cohort	*P*		External validation cohort		*P*
	Alive	Dead		Alive	Dead		Alive	Dead	
	*N* = 163	*N* = 668		*N* = 55	*N* = 301		*N* = 21	*N* = 42	
Age									
< 60	88(53.98)	265(39.67)	0.001	31(56.36)	111(36.88)	0.007	14(66.67)	8(19.05)	< 0.001
≥60	75(46.02)	403(60.33)		24(43.64)	190(63.12)		7(33.33)	34(80.95)	
Sex									
Male	84(51.53)	358(53.69)	0.637	25(45.45)	165(54.82)	0.202	11(52.38)	23(54.76)	0.861
Female	79(48.47)	310(46.41)		30(54.55)	136(45.18)		10(47.62)	19(45.24)	
Tumor location									
Colon	111(71.78)	544(81.44)	0.001	44(80.00)	250(83.06)	0.584	12(57.14)	39(92.86)	< 0.001
Rectum	52(28.22)	124(18.56)		11(20.00)	51(16.94)		9(42.86)	3(7.14)	
Differentiation									
Well	7(4.29)	24(3.59)	0.160	2(3.63)	13(4.32)	0.162	4(19.05)	1(2.38)	0.245
Moderately	123(75.46)	444(66.47)		44(80.00)	210(69.77)		13(61.90)	29(69.05)	
Poorly	22(13.50)	147(22.01)		7(12.73)	56(18.60)		3(14.29)	10(23.36)	
Undifferentiated	3(1.85)	35(5.24)		2(3.64)	17(5.65)		0(0)	1(2.38)	
Unknow	8(4.90)	18(2.69)		0(0)	5(1.66)		1(4.76)	1(2.38)	
Histological type									
Adenocarcinoma	147(90.18)	579(86.68)	0.619	51(92.73)	261(86.72)	0.694	19(90.48)	37(88.10)	0.574
Mucinous adenocarcinoma	6(3.68)	48(7.19)		0(0)	20(6.64)		1(4.76)	5(11.90)	
Signet ring cell carcinoma	0(0)	2(0.30)		0(0)	1(0.33)		0(0)	0(0)	
Other	10(6.14)	39(5.83)		4(7.27)	19(12.31)		1(4.76)	0(0)	
Size, mm									
< 55	100(61.35)	328(49.10)	0.005	35(63.64)	161(53.49)	0.165	17(80.95)	25(59.52)	0.072
≥ 55	63(38.65)	340(50.90)		20(36.36)	140(46.51)		4(19.05)	17(40.48)	
TNM stage									
IVB	163(100)	668(100)		55(100)	301(100)		21(100)	42(100)	
T1	2(1.23)	3(0.45)		0(0)	0(0)		0(0)	0(0)	0.075
T stage									
T2	6(3.68)	13(1.95)		0(0)	4(1.33)		0(0)	0(0)	
T3	108(66.26)	352(52.69)		34(61.82)	152(50.50)		15(68.12)	20(47.62)	
T4	47(28.83)	300(44.91)		21(38.18)	145(48.17)		6(31.88)	22(52.38)	
N0	32(19.63)	78(11.68)	< 0.001	7(12.73)	26(8.64)	0.029	3(14.29)	2(4.76)	0.001
N stage									
N1	78(47.85)	233(34.88)		27(49.09)	109(36.21)		4(19.05)	5(11.90)	
N2	53(32.52)	357(53.44)		21(38.18)	166(55.15)		14(66.66)	35(83.34)	
M stage									
M1a	0(0)	0(0)	–	0(0)	0(0)	–	0(0)	0(0)	–
M1b	163(100)	668(100)	1	55(100)	301(100)	1	21(100)	42(100)	1
Radiotherapy									
Yes	29(17.79)	31(4.64)	< 0.001	2(3.64)	9(2.99)	0.800	4(19.05)	2(4.76)	0.071
None/Unknown	143(82.21)	637(95.36)		53(96.36)	292(97.01)		17(80.95)	40(95.24)	
Chemotherapy									
Yes	150(92.02)	446(66.77)	< 0.001	48(87.27)	194(64.45)	0.001	17(80.95)	24(57.14)	0.047
None/Unknown	13(7.98)	222(33.23)		7(12.73)	107(35.55)		4(19.05)	18(42.86)	
No. of sampled LNs Median (IR)	16(11)	16(10)	0.219	16(10)	16(10)	0.821	20(9)	16(9)	0.031
No. of Positive LNs Median (IR)	2(5)	4(7)	< 0.001	4(5)	3(7)	0.001	2(5)	2(7)	0.570
Primary surgery									
Yes	163(100)	667(99.85)	0.622	53(100)	300(99.67)	0.670	21(100)	41(97.62)	0.484
No	0	1(0.15)		0	1(0.33)		0(0)	1(2.38)	
Metastatic surgery									
Yes	49(30.06)	118(17.66)	< 0.0001	11(20.00)	59(19.60)	0.946	5(23.81)	4(9.52)	0.131
No	114(69.94)	550(82.34)		44(80.80)	242(80.40)		16(76.19)	38(90.48)	
CSS									
Yes	91	491	0.211	25	217	0.236	11	37	0.025
No	72	177		30	84		10	4	

### Independent prognostic factors in patients with CRCHPM

**Fig. 2:**
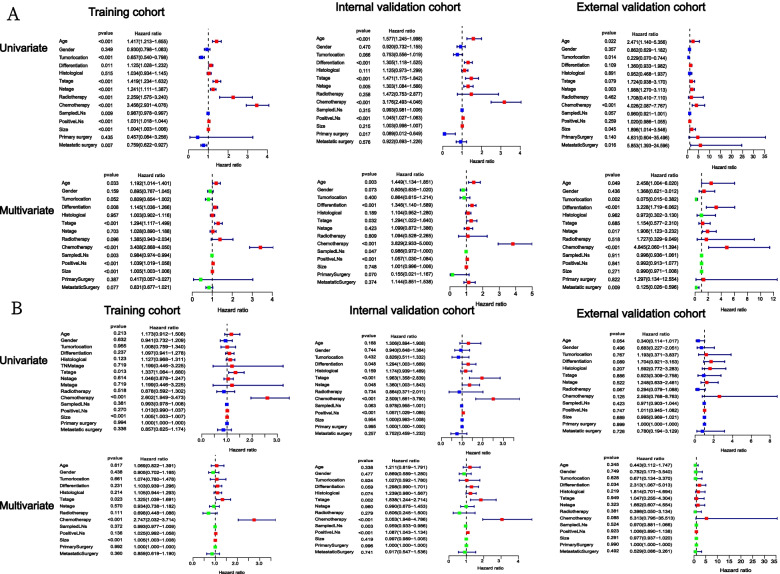
Univariate and multivariate analysis in three cohorts. Univariate and multivariate cox regression models are used in OS training, internal validation, and external validation cohorts (**A**). Univariate and multivariate cox regression models are used in CSS training, internal validation, and external validation cohorts (**B**)

According to univariate cox regression analysis of the training cohort, age, tumor location, degree of differentiation, T stage, N stage, tumor size, radiotherapy, chemotherapy, number of sample lymph nodes, number of positive lymph nodes were closely related to OS. Multivariate cox regression analysis showed that age, degree of differentiation, T stage, chemotherapy, number of sample lymph nodes, number of positive lymph nodes and tumor size were independent prognostic factors of CRCHPM. In the internal validation cohort, univariate cox analysis indicated that age, degree of differentiation, T stage, N stage, chemotherapy, number of sample lymph nodes, number of positive lymph nodes, Primary surgery was closely related to OS. Multivariate cox analysis showed that age, degree of differentiation, T stage, chemotherapy and number of positive lymph nodes were independent prognostic factors of CRCHPM. Multivariate analysis in an external validation cohort suggested that age, tumour location, degree of differentiation, N stage, chemotherapy and Metastatic surgery were independent prognostic factors (Fig. 2A).

In the CSS study, univariate and multivariate cox regression analysis of the training cohort indicated that T stage, chemotherapy, and tumor size were independent prognostic factors. Univariate and multivariate cox analysis of internal validation cohort indicated that T stage, chemotherapy and number of positive lymph nodes were independent prognostic factors. Externally verified multivariate analysis suggested that the degree of differentiation was an independent prognostic factor (Fig. 2B).

### Construction of the nomogram

**Fig. 3:**
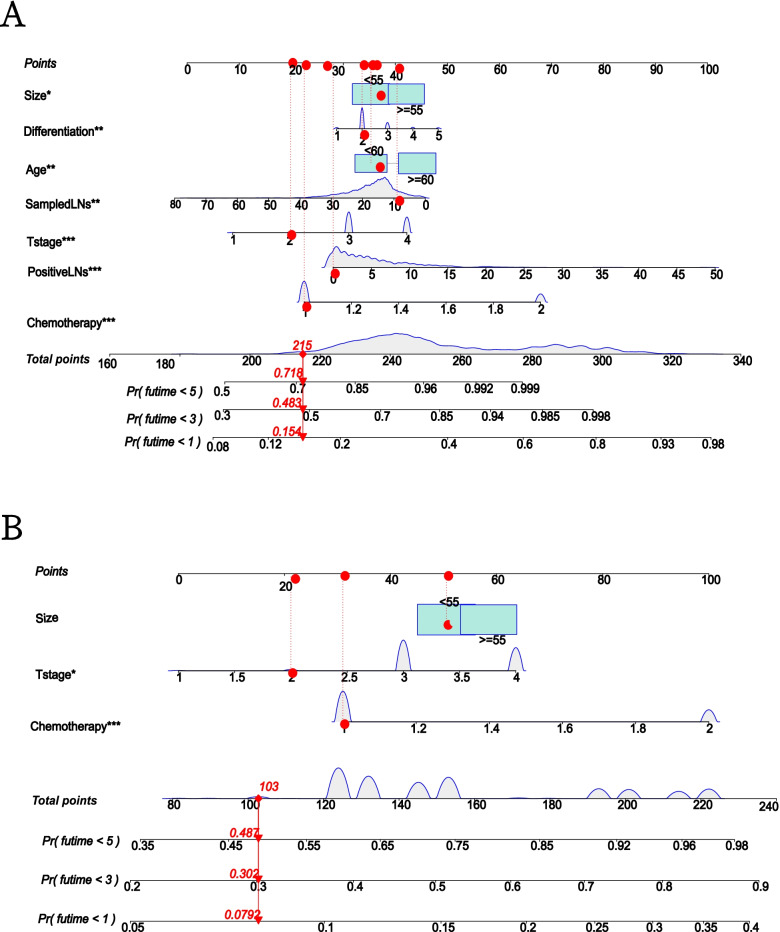
Nomogram for predicting CRCHPM patient outcomes. Assessment of overall survival (OS) and cancer-specific survival (CSS) related rolograms in patients with colorectal hepato-pulmonary metastasis (CRCHPM). Establish OS (**A**) and CSS nomogram according to multivariate cox regression analysis of training cohort (**B**)

Develop a nomogram of OS and CSS based on independent prognostic factors of OS and CSS determined by multivariate cox regression of training cohort. According to this nomogram, we can obtain the risk score of each variable, and then add the risk score of each variable to get the total score to obtain the probability of CSS and OS at 1, 3, and 5 years for each patient (Fig. 3).

### Comparison of nomogram with TNM stage and chemotherapy

TNM stage and adjuvant chemotherapy are important indicators to evaluate the prognosis of tumor metastasis. We found that the TNM stage of CRCHPM patients was IVB, and most of the patients with IVB received adjuvant chemotherapy. To confirm whether nomogram can predict the prognosis of CRCHPM patients better than TNM stage and chemotherapy, time-dependent ROC analysis was performed at 1, 3 and 5 years, respectively.

**Fig. 4:**
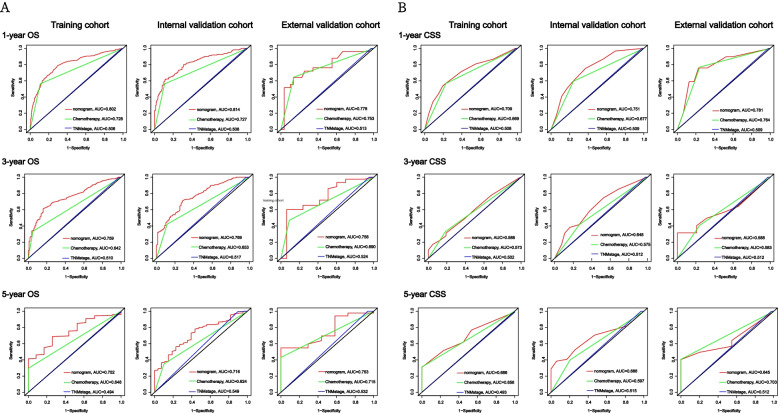
ROC curves analysis. Receiver operating characteristic (ROC) curves of TNM stage, chemotherapy and the nomogram of the OS and CSS. The AUC values of the ROC curve predicted OS and CSS rates for nomogram, TNM stage and chemotherapy at 1, 3, and 5 years in the training cohort, internal validation cohort, and external validation cohort. OS (**A**), CSS (**B**)

The 1-, 3-, and 5-year AUC values of the nomogram for the prediction of OS in the training cohort were 0.802, 0.759 and 0.752, respectively, for chemotherapy were 0.728, 0.642 and 0.648, respectively, and for TNM stage were 0.506, 0.510 and 0.494, respectively. The AUC values of the nomogram predicted 1-, 3- and 5-year OS in the internal validation cohort were 0.814, 0.769, and 0.716, respectively, for chemotherapy were 0.727, 0.653, and 0.624, respectively, and for TNM stage were 0.508, 0.517, and 0.549, respectively. The AUC values of nomogram 1-, 3-, and 5 years of OS predicted in the external validation cohort were 0.778, 0.756, and 0.753, respectively, for chemotherapy were 0.753, 0.690, and 0.715, respectively, and for TNM stage were 0.513, 0.524, and 0.532, respectively (Fig. 4A).

In addition, the 1-, 3-, and 5-year AUC values of the nomogram for the prediction of CSS in the training cohort were 0.709, 0.588, and 0.686, respectively, for chemotherapy were 0.669, 0.572, and 0.656, respectively, and for TNM stage were 0.508, 0.502, and 0.493, respectively. In the internal validation cohort, the 1-, 3-, and 5-year AUC values of the nomogram for the prediction of CSS were 0.751, 0.648, 0.666, respectively, for chemotherapy were 0.677, 0.575, 0.579 respectively, and for TNM stage were 0.509, 0.512, 0.515.The nomogram AUC values predicted for 1, 3, and 5 years of CSS by external validation coenets were 0.781, 0.588, and 0.645, respectively, and the chemotherapy were 0.764, 0.583, and 0.703, respectively, and the TNM stages were 0.509, 0.512, and 0.512, respectively (Fig. 4B).

The ROC curve represents the discrimination ability of nomogram. We found that nomogram showed good sensitivity in predicting OS and CSS at 1, 3, and 5 years in the training cohort, internal validation cohort, and external validation cohort, and was significantly better than TNM stage and chemotherapy (Fig. 4).

### Evaluation and validation of prediction OS and CSS nomogram

**Fig. 5:**
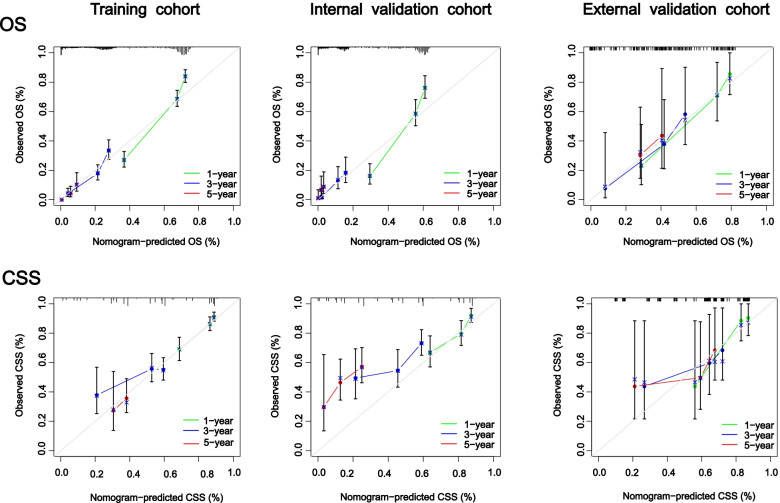
Calibration curves. Calibration curves for 1 -, 3 -, and 5-year CSS and OS rates predicted by nomogram for training, internal validation, and external validation cohorts

C-index values and calibration curves are usually used to evaluate the discrimination of nomogram. In the training cohort, internal validation cohort, and external validation cohort, The c-indexes for predicting OS were 0.719 (95%CI 0.698, 0.740), 0.733 (95%CI 0.702, 0.763), and 0.737 (95%CI 0.661, 0.813), respectively. In addition, the c-indexes of CSS prediction were 0.678 (95%CI 0.632, 0.723), 0.739 (95%CI 0.685, 0.793) and 0.722 (95%CI 0.654, 0.824) in the training cohort, internal verification cohort and external verification cohort, respectively. This indicated that nomogram has a good ability to identify CRCHPM patients. In addition, nomogram’s calibration curve has no obvious deviation from the reference line in the three cohorts, showing high reliability (Fig. 5).

**Fig. 6:**
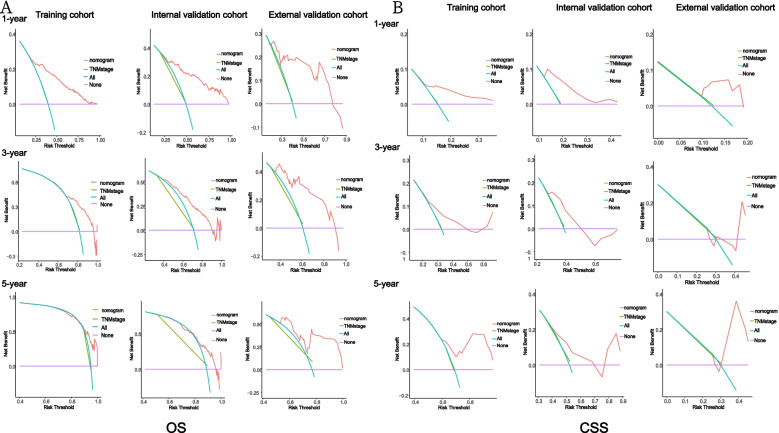
Decision curve analysis. Decision curve analysis of the nomogram and TNM stage for the cancer-specific survival (CSS) and overall survival (OS) prediction of patients with colorectal hepato-pulmonary metastasis (CRCHPM). **A** OS; (**B**) CSS

It is a new method to evaluate the diagnostic accuracy of diagnostic models, which is superior to AUROC in clinical value evaluation. In training, internal validation and external validation cohorts, the nomogram DCA score has a higher net benefit than the TNM stage, indicating that it has better clinical value than the TNM stage (Fig. 6).

## Discussion

CRC is the second most lethal neoplastic disease in the world [25]. The prognosis of colorectal cancer is still unsatisfactory, despite the rapid development of treatment strategies in recent years [26–28]. Patients with metastatic CRC had worse survival outcomes compared with patients without metastases, with a 5-year survival rate of only 14.0% [29]. At present, there are many studies on the distant metastasis of CRC mainly focusing on the CRC liver metastasis. Although the incidence of CRCHPM or multiple metastases is low, we found that the CRCHPM prognosis is worse than liver metastases. There is no study can effectively predicted the prognosis of CRCHPM. We combined the SEER database with our medical center data on the prognosis of CRCHPM patients to identify independent clinicopathological predictors by univariate and multivariate cox analysis and to construct a nomogram to predict CSS and OS. And the results showed that our nomogram based on simple clinicopathological characteristic can accurately predict the 1-year, 3-year, 5-year OS and CSS of CRCHPM patients. At the same time, we used DCA to compare the predictive ability of nomogram with TNM Stage and chemotherapy, and the results showed that our nomogram had a higher predictive ability for the prognosis of CRCHPM than TNM stage and chemotherapy.

There are also relevant literature reports on the prognosis prediction of patients with distant metastasis of colorectal cancer. Liu et al. [30] constructed a nomogram based on 14 factors to predict OS for distant metastasis of CRC, the nomogram was a good predictor of survival in patients with stage IV CRC, but the study was deficient in predicting only OS and lacked an external validation cohort, and did not compare the predictive effect of nomogram with individual factors such as TNM stage and chemotherapy. Tang et al. [19] established nomogram based on 14 prognostic factors of synchronous liver metastasis, and the study found that bone and lung metastases were positively correlated with the risk of synchronous liver metastases and closely related to OS. But the same shortcomings of this study are the lack of an external validation cohort. Wu et al. [31] established nomogram based on 9 factors to predict the OS and CSS of CRC patients with liver metastases only. The study found that compared with the TNM stage system, the constructed nomogram had better prediction performance in predicting OS and CSS. However, this study lacked external validation and the value of C-index of the training and validation sets was not high. Mo et al. [32] used nomogram to predict the OS of metastatic CRC when they predicted the metastatic location of CRC. The results showed that the predictive ability of nomogram for OS and CSS is better than TNM stage were consistent with our research. The conformation of a nomogram based on appropriate clinicopathological characteristic can predict the prognosis of patients with distant metastases in CRC. However, most studies also did not have an external validation cohort and did not predict important prognostic indicators such as CSS. In addition, there are similar results yielded by several studies which are only on OS or single metastases and lack external validation cohort [33–35]. Our model based on clinicopathological characteristic can effectively predict the prognosis of CRCHPM, which has important clinical significance.

Our study found that T stage, tumor size and chemotherapy were critical for the prognosis of CRCHPM. A study showed that the larger the tumor is, the less likely patients will have a good prognosis and the greater risk they have liver, lung and brain metastases from tumor [32]. However, other studies have found the opposite. Li et al. [36] retrospectively studied the relationship between the size of resectable tumors at different sites and prognosis. Their research showed that smaller tumors were associated with poorer OS, CSS, and DFS. Further studies are needed on tumor size and prognosis. Campbell et al. [37] combined T-stage and venous invasion to predict postoperative prognosis in patients with CRC. Univariate analysis showed that T-stage was associated with poorer OS and CSS in patients. Li et al. [38] showed that both T and N stages significantly affected OS in patients with CRC, but the weight of T stage was greater than that of N stage. Wu et al. [39] studied the relationship between early T stage and prognosis in patients with liver metastases from colorectal cancer. The results showed that the prognosis of patients with T1 stage metastasis was significantly lower than that of T3/T4 stage metastasis patients, and early T stage was an independent prognostic factor for low survival rate. Chemotherapy is one of the most common treatment options for patients with CRC metastases. Studies show that in patients with stage II colorectal cancer, regardless of prior treatment, patients’ age, or high-risk pathological risk characteristics, improved OS is associated with adjuvant chemotherapy [40]. In a retrospective study with a median follow-up of 37.0 months, the OS rates of CRC patients with and without adjuvant chemotherapy were 62.1 and 40.4%, respectively [41]. This is consistent with our findings. However, some studies have also shown that adjuvant chemotherapy for CRC patients with lung metastases has no significant effect on OS [42–44]. The benefit of chemotherapy in patients with CRC metastases requires further study. The inclusion of appropriate clinicopathological indicators has important clinical significance in predicting the prognosis of CRCHPM.

However, this study still has some limitations. One is the small sample size of our external validation cohort, which is mainly due to the low incidence of CRCHPM. At the same time, three cohorts had some data missing and could not be included in the analysis. Second, this is a retrospective study based on limited clinical records, and further multicenter prospective clinical studies are needed to demonstrate the clinical validity of this model. Third, we only include clinicopathological characteristics, but also include molecular pathological characteristics (KRAS, BRAF, Mismatch repair/microsatellite instability) in variable analysis to further improve the prediction effect of nomogram. However, our study is also clinically important because there are very few studies to predict the prognosis of CRCHPM patients, and we are the first to propose the application of clinicopathological characteristics to predict OS and CSS with good predictive effect. In addition, we have our own large cohort data from which we screened CRCHPM patients with external validation from our own large sample of more than 3000 patients, making our study more credible. Our study developed an adjunct model to predict the prognosis of CRCHPM, but this adjunct should be used with caution based on the patient’s overall situation.

## Conclusions

In conclusion, we establish and validate a nomogram to predict CSS and OS in patients with CRCHPM based on significant clinicopathological characteristics. In addition to its excellent predictive power, this novel nomogram has sufficient discriminatory and calibration capabilities, it may provide valuable prognostic and predictive information to aid treatment strategies for CRCHPM patients.

## Data Availability

The original contributions presented in the study are included in the article/Supplementary Material. The datasets generated during and/or analysed during the current study are available from the corresponding author on reasonable request.

## Abbreviations

CRC: Colorectal cancer
OS: Overall survival
CSS: Cancer-specific survival
CRCHPM: Colorectal cancer hepato-pulmonary metastasis
WUHCC: Wuhan Union Hospital Cancer Center
AUROC: Area under the receiver operating characteristic curve
C-index: Concordance index
DCA: Decision curve analysis
HR: Hazard ratio
